# The Portuguese version of the visual vertigo analog scale

**DOI:** 10.1016/j.bjorl.2022.09.004

**Published:** 2022-10-13

**Authors:** Ana Margarida Amorim, João Simões, Joana Gonçalves, Mafalda Ferreira, João Carlos Ribeiro

**Affiliations:** a Coimbra University Hospital Centre, Department of Otorhinolaryngology, Coimbra, Portugal; b Centre Hospitalier de Mouscron, Department of Otorhinolaryngology, Réseau Santé, Louvain, Belgium; c University of Coimbra, Faculty of Medicine, Portugal

**Keywords:** Visual vertigo, Visual analog scale, Translation, Validity, Reliability

## Abstract

**Objectives:**

Visual vertigo occurs after a vestibular disorder compromising daily living. It can be assessed by “the Visual Vertigo Analogue Scale” (VVAS), a self-administered questionnaire without Portuguese version. To perform the translation, cross cultural adaptation, and validation of VVAS from English to Portuguese.

**Methods:**

Prospective study involving the translation and cross-cultural adaptation of the VVAS into the Portuguese language, according to recognized guidelines. It was completed by 63 healthy controls and 198 participants with vestibulopathy who also completed the Dizziness Handicap Inventory (DHI) to further explore the link between DHI and VVAS. Groups were compared for severity of visual vertigo and VVAS reliability and internal consistency were tested.

**Results:**

The VVAS score was significantly higher in vestibular group (*p* < 0.001). A Cronbach’s α of 0.9 confirmed the valid internally consistent of the applied version. The severity score of VVAS showed a positive strong correlation with DHI (*p* < 0.0001).

**Conclusion:**

The present Portuguese translation of the scale showed satisfactory properties for the assessment of self-perceived and severity of visual vertigo in a significant group of vestibular Portuguese patients.

**Level of evidence:**

2.

## Highlights

• Visual vertigo occurs after a vestibular disorder compromising daily living.
• Visual vertigo analog scale is reliable to be used in the Portuguese population.
• Visual vertigo is severe across all kinds of vestibular syndromes.

## Introduction

Visually Induced Vertigo (VIV) is usually triggered by a complex, distorted, large field or moving visual stimulus, including the relative motion of the visual surround associated with body movement.^1^ It is an inappropriate response to motion of the visual environment due to overreliance or misinterpretation of visual cues. It has long been recognized consequently to a vestibular insult.^2^ After an initial period of recovery of a few weeks, dizzy symptoms do not fully disappear and are aggravated by looking at moving or repetitive images.^3^ In a recent longitudinal study 4% of all patients between the ages of 18 and 64 years registered with a general practitioner reported persistent and frequent symptoms of dizziness, and at least 3% were severely incapacitated by the symptoms, 18-months later.^4^ This has a major impact in daily living quality of life.^5^

Several questionnaires have been used to measure the quality-of-life impact of VIV. Dizziness Handicap Inventory (DHI) is used to determine the impact of dizziness on various aspects of activities of daily living. Each question is answered on an ordinal scale marking how that situation most applies to the patient. It does not specifically target visual vertigo but is a widely available and used tool for reported outcome measure in vestibular patients,^6^ including a Portuguese version.^7^

The Visual Vertigo Analogue Scale (VVAS) is a quick self-administered, valid, and reliable questionnaire that serves to assess visual vertigo. It has been validated in 102 vestibulopathy group of patients.^8^ It consists of nine visual analog scales subjects’ patients were asked to rate. Each scale pertained to a specific visual vertigo provoking situation. It has been validated into Spanish to use in Argentina.^9^ There is no Portuguese version of this questionnaire, and the authors propose to fill this gap in daily vestibular patients’ evaluation with a clinical, simple, and quickly answerable scale.

Our main objective is to perform the translation, cross cultural adaptation and validation of “Visual Vertigo Analogue Scale” from its ‘original version in English to Portuguese.

## Methods

The study was done in accordance with the principles of the International Conference on Harmonization Good Clinical Practice guidelines and approved by the Research Ethics Committee (reference 351/CES). The study is conformed to Directive 2010/63/EU.

### Translation and cross-cultural adaptation of the VVAS

Prospective study involving the translation and cross-cultural adaptation of the Visual Vertigo Analogue Scale (VVAS) into the European Portuguese language and culture, based in the recommendations of Beaton et al.^10^ STROBE statement checklist was used. Prior consent was requested and obtained from the original author of the VVAS for the development of this instrument.

**Figure 1:**
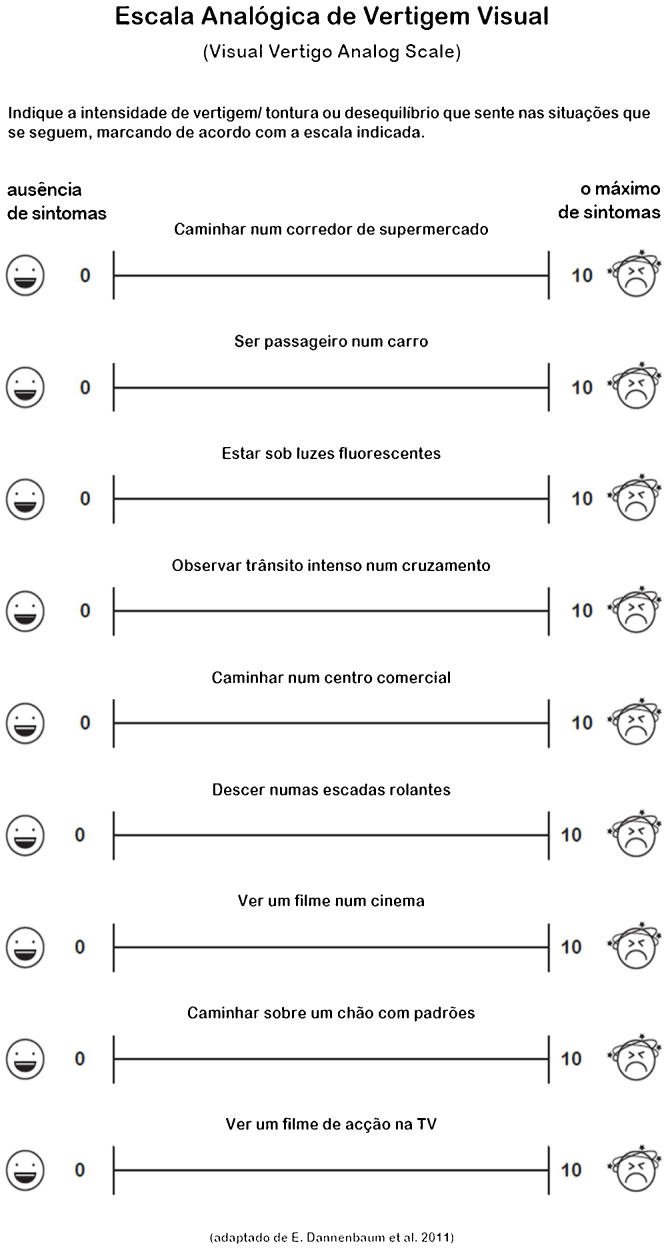
Visual vertigo analog scale Portuguese translation.

The steps of this process were as follows: first, the English to Portuguese translation was performed by one ENT physician expert in vestibular diseases and a naïve translator, who both have Portuguese as their first language. The translators carefully followed the design principles of the original version. Second, this translation was consolidated. Third, a “back translation” from Portuguese into English was performed by one native English individual that was unaware of the original wording. This step was necessary in order to bring out unclear wording or cultural peculiarities in the symptom’s description, and to assure a consistent translation of the content of each item. Fourth, the back translation was checked against the original wording to ensure that each translated item captured the nuances of the original English wording. If there had been differences in nuance between the original version and the back translated version, the translation would have been modified to improve the correspondence between the two versions, but this final procedure was not necessary. Prior to these versions conference, Elizabeth Dannenbaum (author of the original VVAS) compared the original VVAS with both back translations. Finally, the complete translation included not only the items but also the instructions and the overall format of the questionnaire. All of these were carefully reproduced from the English original to produce an accurate translation (Fig. 1).

### Subjects or participants

Two groups were recruited. The vestibulopathy group consisted of 198 subjects who were sequentially enrolled at our tertiary outpatient Equilibrium Clinic from December 2017 to December 2019. Each subject was diagnosed by one experienced Otolaryngologist based in a structured interview, physical examination, and complementary exams as necessary according to the International Classification of Vestibular Disorders from the Barany’s Society.^11^ The control group consisted of 63 healthy escorts consecutively extracted from the same population without history of vestibulopathy symptoms or any other otological disease that also volunteered to answer the questionnaire. Both groups volunteered to answer the 9-item scales and the DHI before the appointment. The participants in the study did not have any help from the assistant and the answers were reviewed for completeness.

### Measurement questionnaires

Portuguese VVAS consists of nine visual analog scales which subjects were asked to rate. Each scale pertained to a specific visual vertigo provoking situation. The subjects should mark a vertical line on a 10-cm line to indicate the amount of dizziness provoked by each situation, between two anchors, with zero (0) representing no dizziness and ten (10) representing an extreme dizziness or activity avoided due to dizziness. The distance from the zero anchor to the subject’s marking was measured to the nearest next whole number in centimeters. Items that were not applicable to the participant’s daily life (e.g., going to movies) were not completed. If the subject answered with a whole number, that was the one that was considered. To summarize the nine items, two final scores were calculated: Visual Vertigo Analogue Scale (VVAS) positive and Visual Vertigo (VV) severity.^8^

The subjects were classified as VVAS positive if two or more items were rated above zero on the analogue scale. A parameter of interest was the VV severity score that was calculated as an average based on the mean scores of non-missing items: VV Severity = (rated analogue scale items/number of answered items) × 10. Thus, a VV severity score of 0 indicated that the subject did not experience Visual Vertigo, whereas a score of 90–100 will indicate severe visual vertigo.^8^

The DHI is a 25-item questionnaire that asks the patients to rate their self-perception of disability from dizziness. DHI consists of a 7-item physical subscale, a 9-item emotional subscale, and a 9-item functional subscale. A score of 4-points is assigned to a “yes” response, 2-points to “sometimes”, and 0-points to “no” response. Thus, the total score ranges from 0 (no perceived disability) to 100 (maximum perceived disability). Scores can fluctuate from normal (less than 15 points), mild handicap (16–34 points), moderate handicap (36–52 points) or severe (>53 points).^12^

### Statistical analysis

Data collected were analyzed using SATA 15®. The sample was described in terms of the distribution of the descriptive variables by means of summary measures.

To compare the proportion of positive VVAS in control and vestibular groups, Chi-Square statistics was applied. A Mann–Whitney test for independent samples was carried out to compare VV severity scores between control and vestibular groups. Test-retest reliability of the positive VVAS was assessed with the Wilcoxon test. The internal consistency of the VVAS was examined by the Cronbach’s alpha statistical index.

The VV severity score was compared between the controls and vestibular subgroups resorting to a Kruskal Wallis test. Post-hoc comparisons were performed using Bonferroni correction. A Spearman correlation analysis was carried out to explore relationship between VV Severity and DHI total scores. The statistical significance was set at 0.05.

## Results

### Subjects

The VVAS was tested in 198 participants with vertigo and 63 healthy subjects. The mean age of the vestibulopathy group was 58.0 ± 13.3 years old with 65.7% (130/198) being females. The control group consisted of 63 subjects with 43.4 ± 13.3 years old, where 44.4% (23/63) were females. Table 1 resumes characteristics of participants.

**Table 1:** Characteristics of the participants.

Characteristics	Vestibulopathy group (n = 198) (%)	Subgroup for reliability testing (n = 45) (%)	Controls (n = 63)
Age in years; mean (SD)	58.0 (13.3)	60 (12)	43.4 (13.3)
Gender n women/men (% women)	130/68 (65.7)	31/14 (68.9)	23/45 (44.4)
Ménière disease	42 (16.1)	14 (13.1)	–
Benign paroxysmal positional vertigo	33 (12.6)	8 (17.8)	–
Acute unilateral vestibulopathy	26 (10)	4 (8.9)	–
Chronic unilateral vestibulopathy	22 (8.4)	3 (6.7)	–
Bilateral vestibulopathy	5 (1.9)	2 (4.4)	–
Vestibular migraine	25 (9.6)	6 (13.3)	–
Central vestibulopathy	8 (3.1)	–	–
Persistent postural perceptual dizziness	17 (6.5)	4 (8.9)	–
Benign recurrent vestibulopathy	6 (2.3)	2 (4.4)	–
Mix group^a^	12 (5.4)	2 (4.4)	–

a Mix group includes: multifactorial disequilibrium, semicircular canal dehiscence syndrome, post otitis labyrinthitis, post stapedotomy vertigo, cerebellopontine angle arachnoid cyst, vestibular schwannoma, post traumatic labyrinthitis.

### VVAS characteristics

The VVAS was positive in 170 patients (85.9%) and in 10 (15.9%) subjects in the control group. A statistically significant association between VVAS positive and vestibulopathy group patients was found (*p* < 0.001) (Table 2). Visual vertigo scores were significantly different between control and vestibulopathy groups, respectively (2.3 ± 0.9 vs. 35.3 ± 1.8, *p* < 0.001).

**Table 2:** Proportion of VVAS positive and negative subjects in the control and vestibular groups.

	Visual vertigo analog scale		*p*
	Positive	Negative	
Vestibulopathy group	170	28	<0.001
Control group	10	53	<0.001
Total	180	81	<0.001

### Internal and external reliability

The Cronbach’s Alpha analysis of 0.95 indicated that the VVAS was internally consistent and reliable. Moreover, item-to-total correlation coefﬁcients revealed moderate-to high associations between VVAS individual items and total score, further evidencing homogeneity and strong internal consistency of the VVAS.

For test-retest reliability estimations, 45 patients repeated the VVAS (7–658, 291) days. No statistically significant differences between the results of the initial positive VVAS and the final positive were found (*p* > 0.05).

### VV severity between controls and the vestibulopathy group

VV severity scores were significantly different between patients and controls (*p* < 0.001). Post hoc tests reveal the existence of statistically significant differences between control subjects and patients with Persistent Postural Perceptual Dizziness (PPPD), Ménière Disease (MD), Vestibular Migraine (VM), Chronic Unilateral Vestibulopathy and Acute Unilateral Vestibulopathy and Benign Paroxysmal Positional Vertigo (BPPV) (*p* < 0.001), as well as with Central Vestibulopathy, Bilateral vestibulopathy and Semicircular Dehiscent Syndrome (*p* < 0.05).

No statistically significant differences were found between controls and subjects of the other groups (*p* = 1.000), nor comparing the subgroups between themselves (*p* > 0.05).

### Correlation between the VVAS and DHI

Non-parametric Spearman correlation analysis carried out between total DHI and VV severity scores for the vestibulopathy patients revealed positive strong correlation (*r* = 0.82, *p* < 0.001).

## Discussion

Visual vertigo analog scale is an important tool to evaluate visual vertigo in vestibulopathy patients.^8^, ^9^ It is a simple tool that discriminates effectively controls from vestibular patients with different etiologies as we could verify with our present work. The positive strong correlation with the DHI that we could demonstrate contributes to the validation of this instrument to be used in the vestibulopathy population. Because of language and cultural differences, it’s original version use is limited in non-validated populations. After a thorough validation to the Portuguese population, we can now use it with confidence and more accurate conclusions in a wide range of patients that present with dizziness symptoms.

In the present study we could find increased levels of visual vertigo severity in PPPD, MD, VM, acute and chronic vestibular syndrome and BPPV groups. This is in agreement with previous studies.^13^, ^14^, ^15^, ^16^, ^17^, ^18^, ^19^, ^20^ Patients suffering from 3PD includes as diagnostic criteria visual vertigo, so our results of higher levels of visual vertigo severity were expected. In fact, the VVAS has got some similar questions also present in a PPPD questionnaire.^13^ A recent study revealed that participants with MD had higher levels of motion sickness susceptibility, visual display dizziness, social life, and work impact of dizziness versus controls.^14^ Patients with VM are reported to be more vulnerable to subjective sensations of dizziness and unsteadiness in challenging motion environments, probably because the compensatory mechanisms could not properly restore higher order perceptual functions. These results are compatible with the previously proposed central sensitization theory.^15^, ^16^ As MD and VM have been considered as a continuum^17^, ^18^ similar results could be accepted in MD patients. In acute and chronic vestibular syndromes increased visual vertigo was described and implicated in the prediction of the development of long-term dizziness.^19^ Increased visual vertigo in our BPPV patients also goes in concurrence with increased visual dependence that has been previously reported in this diagnostic group.^20^

We were not able to match the sociodemographic data from the case group and the control group participants, which may have influenced the results. The time between the test and re-test reliability was not homogeneous but reflects real life conditions. Although it is expected that patients with longer retest times would have lower test-retest reliability as they would feel better because of treatments or natural compensation, it seems that the visual vertigo tends not to disappear.

Further studies are needed to study clinical sensitivity changes of VVAS with specific treatments to correlate visual vertigo severity with vestibular function and visual dependence assessment (for example, by computed posturography or dynamic visual vertical).

## Conclusion

The present Portuguese translation of the VVAS shows good psychometric properties for the assessment of self-perceived and severity of visual vertigo in a large vestibular Portuguese patient group.

## Conflicts of interest

The authors declare no conflicts of interest.
